# Removing Fluoride-Terminations from Multilayered V_2_C*T*_*x*_ MXene by Gas Hydrolyzation

**DOI:** 10.1021/acsomega.2c02441

**Published:** 2022-06-24

**Authors:** Frode Håskjold Fagerli, Zhaohui Wang, Tor Grande, Henning Kaland, Sverre M. Selbach, Nils Peter Wagner, Kjell Wiik

**Affiliations:** † Department of Materials Science and Engineering, NTNU Norwegian University of Science and Technology, Sem Sælands vei 12, NO-7034 Trondheim, Norway; ‡ SINTEF Industry, Richard Birkelands vei 3, NO-7034 Trondheim, Norway

## Abstract

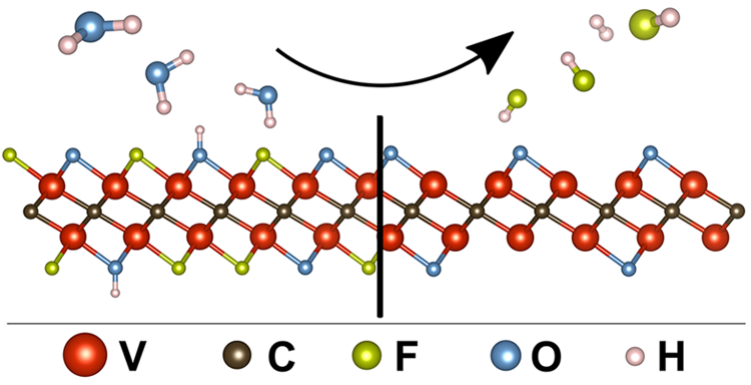

Two-dimensional MXenes have shown great promise for many different applications, but in order to fully utilize their potential, control of their termination groups is essential. Here we demonstrate hydrolyzation with a continuous gas flow as a method to remove F-terminations from multilayered V_2_C*T*_*x*_ particles, in order to prepare nearly F-free and partly bare vanadium carbide MXene. Density functional theory calculations demonstrate that the substitution of F-terminations is thermodynamically feasible and presents partly nonterminated V_2_CO as the dominating hydrolyzation product. Hydrolyzation at elevated temperatures reduced the F content but only subtly changed the O content, as inferred from spectroscopic data. The ideal hydrolyzation temperature was found to be 300 °C, as a degradation of the V_2_C*T*_*x*_ phase and a transition to vanadium oxycarbides and V_2_O_3_ were observed at higher temperature. When tested as electrodes in Li-ion batteries, the hydrolyzed MXene demonstrated a reduced polarization compared with the pristine MXene, but no change in intercalation voltage was observed. Annealing in dry Ar did not result in the same F reduction, and the importance of water vapor was concluded, demonstrating hydrolyzation as a new and efficient method to control the surface terminations of multilayered V_2_C*T*_*x*_ post etching. These results also provide new insights on the thermal stability of V_2_C*T*_*x*_ MXene in hydrated atmospheres.

## Introduction

Since the first report of MXenes 11 years ago,^1^ the research interest for this family of two-dimensional materials has grown exponentially. MXenes are transition metal carbides, nitrides and carbonitrides, where the transition metal “M” and the carbon or nitrogen “X” atoms are stacked in odd numbered layers (e.g., Ti_3_C_2_, V_2_C, and Nb_2_C).^2^ Because of their unique combination of tunable properties, such as metallic conduction, hydrophilic surfaces, adjustable interlayer spacings and rich surface chemistries, MXenes have been reported for a range of applications, from water purification and biomedicine to hydrogen evolution and energy storage.^2−6^ Not only can the combination of M and X elements, or the number of layers, change the properties of the MXene, but with the formation of surface terminal groups upon synthesis, such as -O, -OH, -F, and -Cl, the tuning possibilities of these materials are significant. However, although there have been reported dozens of different MX compositions, there are few methods to fully control the surface terminations on a limited number of MXenes.^6,7^ As the surface terminations determine the local environment in between the MXene layers, and properties such as electronic conduction and ion-intercalation, controlling them is of critical importance for full utilization of MXenes’ potential.^8−11^

Using the most common etching methods for MXene consisting of HF solutions or a solution of HCl and F-salts, a mixture of the above-mentioned termination groups are formed.^12−14^ Although there have been reports on vacuum annealing, oxygen annealing, and treatment in alkalic solutions, where the aim was to change the termination groups post etching, they usually only report on partly termination substitution and often in nonscalable methods such as on thin films.^9,11,15−18^ To prove useful for practical applications such as battery electrodes, the termination groups within the bulk of multilayered MXene particles must be reliably controlled. To the authors’ knowledge, it remains to be demonstrated a method to homogeneously control terminations in MXenes after the etching in F-containing solutions.

An example of a MXene where surface control is important is the V_2_C*T*_*x*_ phase, which is one of the best compositions for electrode materials in supercapacitors and batteries because of its predicted potential.^19−21^ Even though V_2_C*T*_*x*_ already has demonstrated some of the highest capacities of MXenes in Li-ion batteries (LiBs) and supercapacitors,^22,23^ there is still predicted higher capacity, higher voltage, and lower migration barriers in V_2_CO_2_ compared with OH- and F-terminated V_2_C*T*_*x*_.^19,24−27^ However, synthesizing V_2_CO_2_ without decomposing the MXene structure is challenging, because vanadium oxides can form at elevated temperatures by hydrothermal treatment,^28,29^ as well as by annealing in inert, reducing and oxidative atmospheres.^30,31^ Hence, changing the termination groups of V_2_C*T*_*x*_ without formation of secondary phases remains a challenge.

In this work, we demonstrate the use of gas hydrolysis to change the surface terminations of V_2_C*T*_*x*_ MXene. Three different hydrolyzation reactions are proposed, and density functional theory (DFT) calculations support that a shift from F termination to OH/O termination is thermodynamically feasible, given a continuous flow of humidified Ar gas. Therefore, V_2_C*T*_*x*_ particles synthesized by regular HF-etching of V_2_AlC were exposed to a controlled water vapor pressure at various temperatures. X-ray photoelectron spectroscopy (XPS) and energy dispersive X-ray spectroscopy (EDS) were used to verify the chemical change upon hydrolyzation at elevated temperature, indicating a significant reduction of F content upon hydrolyzation at elevated temperatures. X-ray diffraction (XRD), scanning electron microscopy (SEM), and Raman spectroscopy were used to describe the structural changes, showing how the MXene phase remains stable up to 300 °C. These hydrolyzation results are also compared to annealing in dry Ar gas by thermogravimetric analysis (TGA), demonstrating that the water vapor is essential for the resulting change in termination. In the end, galvanostatic cycling of V_2_C*T*_*x*_ electrodes in Li-ion batteries is presented to indicate the change in electrochemical properties of V_2_C*T*_*x*_ upon modifying the MXene surface.

## Results and Discussion

### Thermodynamics

With the intention of substituting F-terminations with O-containing terminations, the following three hydrolyzation reactions are suggested:
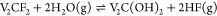
1
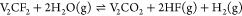
2
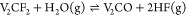
3

**Figure 1:**
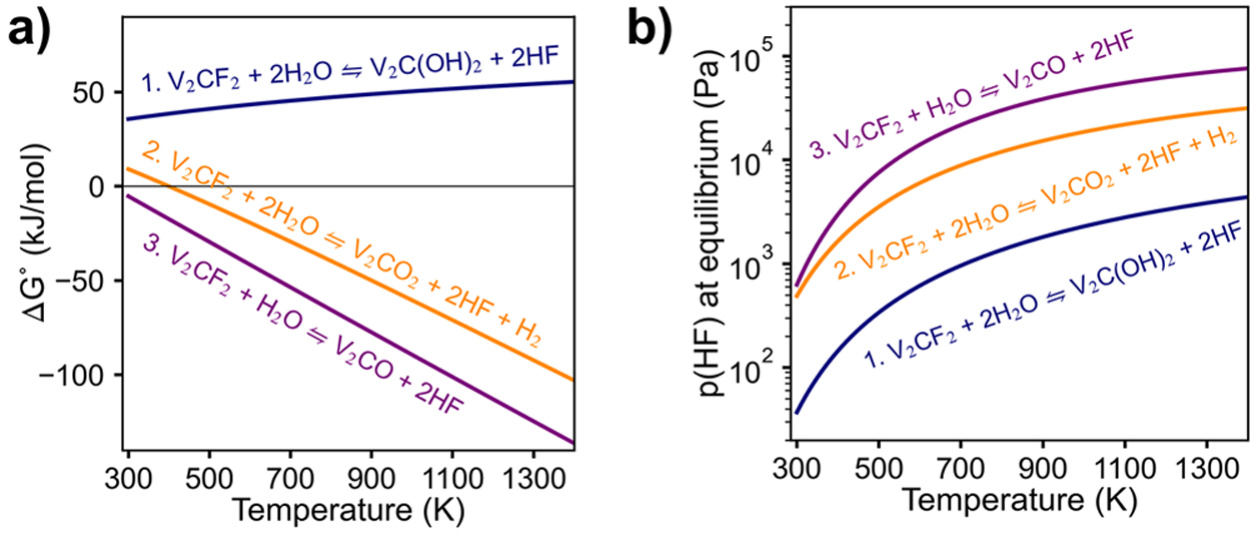
Thermodynamic properties from DFT calculations of three reactions for removal of F-termination from V_2_C*T*_*x*_. (a) shows Δ*G*° as a function of temperature, while (b) shows the equilibrium partial pressure of HF gas for the three reactions, considering a saturated water vapor pressure of 4.738 × 10^4^ Pa.

The variation in standard free energy with temperature for the three hydrolyzation reactions is given in Figure 1a. While reaction 1 remains positive for all temperatures, reaction 3 has a negative Δ*G*° at *T* > RT and reaction 2 at *T* > 400 K, indicating spontaneous reactions at elevated temperatures. However, these results only illustrate the situation at standard conditions, and Figure S11 demonstrates how the Δ*G* of reaction 1 also becomes spontaneous with a sufficiently high ratio between the vapor pressure of H_2_O and HF. In general, having a high vapor pressure of water and continuous removal of HF gas is beneficial for all the presented hydrolyzation reactions. With a given water vapor pressure of 4.738 × 10^4^ Pa, originating from the saturated water gas at 80 °C,^32^Figure 1b presents the calculated equilibrium partial pressures of HF gas for the three reactions and demonstrates that significant amounts of HF gas will be produced at elevated temperatures. It also shows that the dominating reaction would be substitution of two F terminations in favor of a single O-termination, leaving parts of the surface unterminated (V_2_CO). In total, these results give a strong indication that the removal of F-terminations from V_2_C*T*_*x*_ by hydrolyzation is possible, as all the proposed reactions show spontaneous reactions at achievable experimental conditions.

### Structure and Morphology

**Figure 2:**
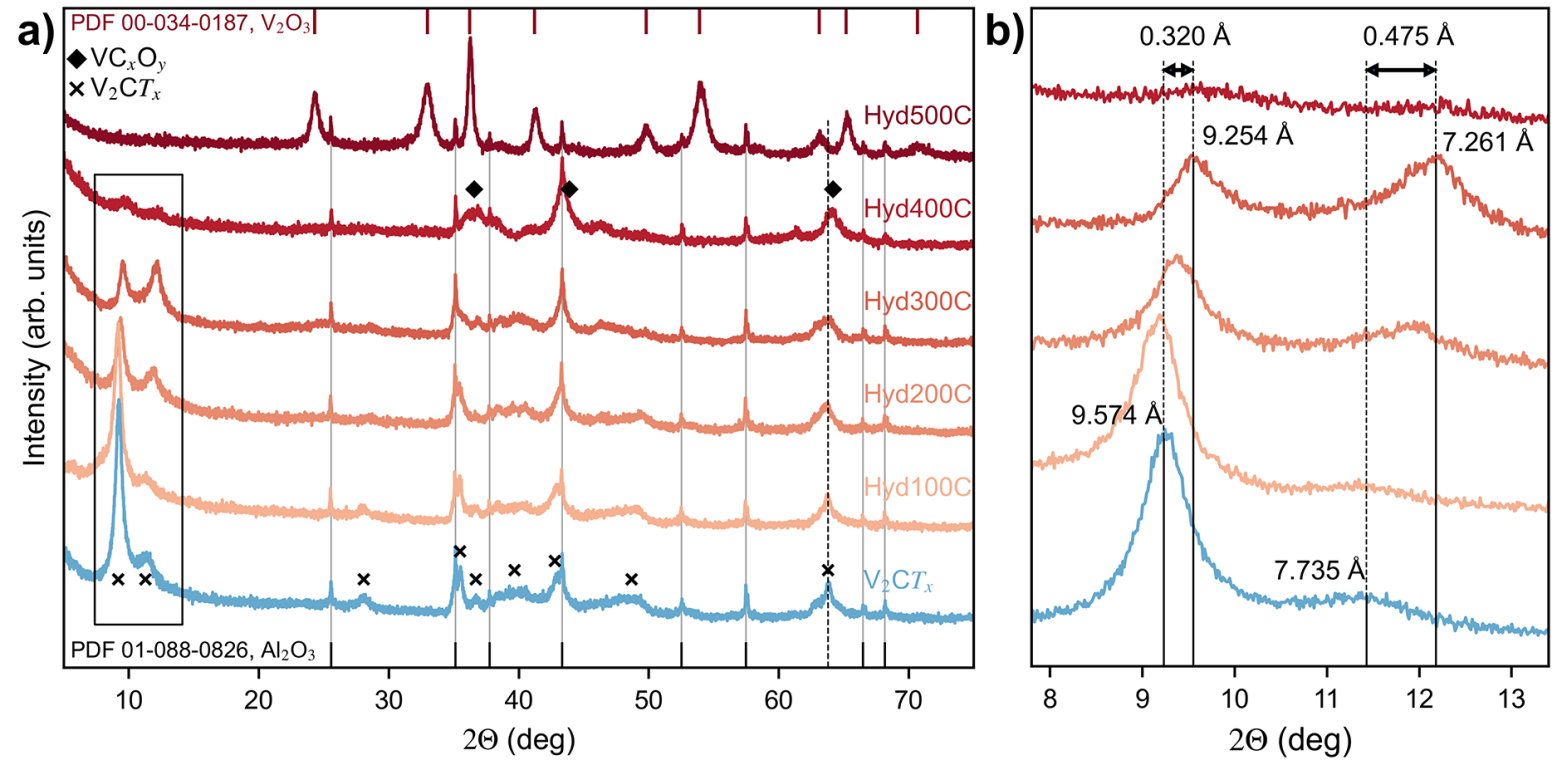
X-ray diffractograms of V_2_C*T*_*x*_ hydrolyzed at different temperatures (a), where (b) shows a close-up of the (002) V_2_C*T*_*x*_ reflections located inside the black rectangle in (a). In (a), there is also a dashed line indicating the position of the (110) MXene reflection at 63.8°.

The structural change of the V_2_C*T*_*x*_ phase upon hydrolyzation is illustrated in Figure 2. It shows that the intensity of the MXene related reflections remain relatively stable up to a hydrolyzation temperature of 300 °C, before they are significantly reduced at 400 °C and virtually absent at 500 °C. The most likely explanation for the degradation at 400 °C can be described by the broad reflections emerging at around 36.6°, 43.5°, and 64.2° which most likely comes from a mixture of vanadium oxycarbides (denoted “VC_*x*_O_*y*_”), as both VC and VO crystallize in the same rock salt structure (space group *Fm*3̅*m*)^33^ and show XRD reflections in those three areas (PDF 01-074-1220 and PDF 04-004-9038). However, these reflections disappear upon increasing the temperature to 500 °C, where the only detectable reflections remaining are related to Al_2_O_3_ from the MAX phase synthesis and V_2_O_3_. This indicates that the V_2_C*T*_*x*_ first decomposes to VC_*x*_O_*y*_ followed by the formation of V_2_O_3_ at temperatures above 400 °C. The transition to V_2_O_3_ after hydrolyzation at 500 °C is also demonstrated by Raman measurements (Figure S5), where the MXene related vibration bands disappear in favor of V_2_O_3_ bands around 210 cm^–1^, and the D and G bands are attributed to amorphous carbonaceous species that remain. For an oxygen terminated MXene phase (V_2_CO_2_), the degradation can be described by the following reaction:
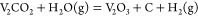
4

However, apart from the degradation at 400 °C significant changes to the MXene phase after hydrolyzation at lower temperatures are observed. First, it should be noted that already in the pristine MXene, the (002) double reflection indicates that two different interlayer spacings are present in the MXene (9.574 and 7.735 Å, Figure 2b). This reflection splitting may be due to intercalation of water molecules in parts of the particles. Xie et al. calculated that the interlayer spacing of V_2_C*T*_*x*_ lies around 7.5 Å with no intercalated water and around 9.5 Å with 2 layers of water molecules, in fair agreement with our experimental results.^34^ Since previous articles often report on an interlayer spacing around 9.5 Å or higher, it is reasonable to assume that parts of our particles have been dried prior to the characterization.^31,35,36^ Interestingly, the split remains after hydrolyzation, although we see a shift in intensity toward the reflection at higher 2θ, indicating a reduction of interlayer spacing and less water between the layers. Additionally, we see an uneven shift of the two reflections toward larger 2θ upon increasing the hydrolyzation temperature. After hydrolyzation at 300 °C, the reduction of the interlayer spacing was 0.320 Å for the reflection at lower 2θ and 0.475 Å for the reflection at higher 2θ (Figure 2b). This change is ascribed to changes in the surface terminations of the MXene, as well as small changes in intercalated water.

Another interesting aspect with the XRD results is that no reflections related to the MAX phase remain after the etching, indicating complete Al removal from the MAX phase (Figure S2). This is usually not reported for HF-etched V_2_AlC and demonstrates that reducing the particle size prior to etching can help improve the etching yield of this phase.^22,31^ With an average size of 5.91 μm (Figure S3), it is shown that 72 h of etching in 48 wt % HF is enough for complete conversion to MXene at ∼22 °C.

**Figure 3:**
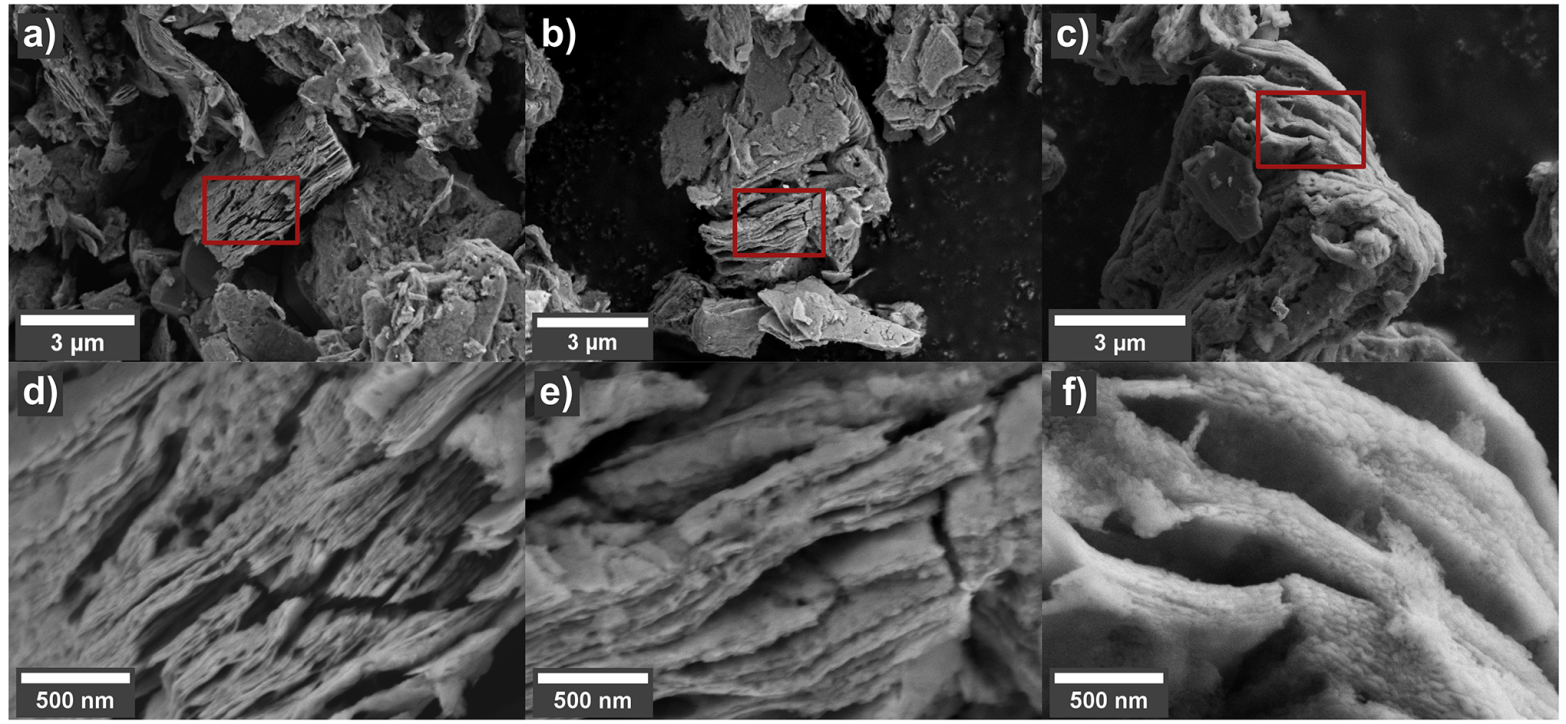
SEM images with low (a–c) and high (d–e) magnification: pristine V_2_C*T*_*x*_ (a,d) and V_2_C*T*_*x*_ hydrolyzed at 300 °C (b,e) and 500 °C (c,f). The high-magnification areas represent the red rectangle in the low-magnification images.

The change in morphology with hydrolysis and temperature is given in Figure 3. Although the macroscopic disc-like morphology remains similar even after the phase transition to V_2_O_3_ (a–c), the high-magnification images (d–f) reveal a formation of nanoparticles at the edges of the particles after hydrolyzation at 500 °C, which most likely represent V_2_O_3_. A similar growth of oxide nanoparticles has been reported after hydrothermal treatment of V_2_C*T*_*x*_, although they report on higher oxidation states of V, corresponding to VO_2_ and V_2_O_5_.^28,29^ In Ti-based MXenes the formation of TiO_2_ nanoparticles at the edges of Ti_3_C_2_*T*_*x*_ and Ti_2_C*T*_*x*_ is also commonly observed upon exposure to water and air at elevated temperatures.^37−40^ The average particle size of the MXenes is a few microns, indicating that the particle size is maintained upon etching, as it matches well with the V_2_AlC MAX phase (Figures S3 and S4). Figure 3 also shows that the particles have a lot of cracks and uneven surfaces. This might originate from introduction of strain in the particles during milling of the MAX phase as it can also be seen in the MAX phase particles before etching (Figure S4).

### Chemical Composition

**Figure 4:**
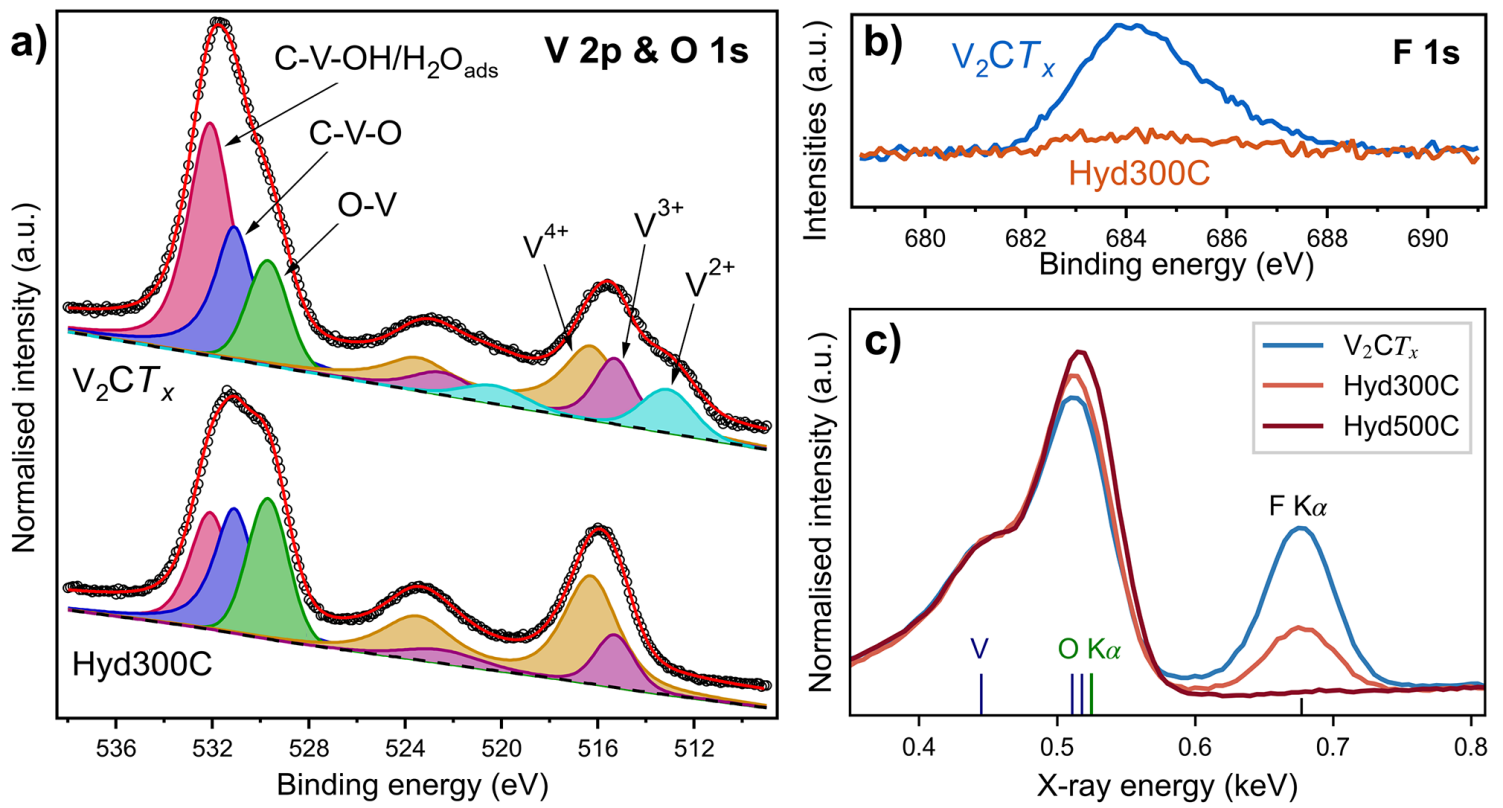
XPS spectra of the V 2p and O 1s region (a) and the F 1s region (b) for V_2_C*T*_*x*_ and V_2_C*T*_*x*_ hydrolyzed at 300 °C, where the intensity of (a) is normalized to the V peaks. (c) The lower energy region of EDS spectra obtained from mapping of V_2_C*T*_*x*_ and V_2_C*T*_*x*_ hydrolyzed at 300 °C and 500 °C, where the intensities are normalized to the V LL peak at 0.446 eV, to show the relative shift of the O and F content.

The change in chemical environment upon hydrolyzation is described in Figure 4, where XPS is used to probe the outer surface of the MXene particles and EDS is used for bulk characterization. Figure 4a presents the deconvolution of the XPS regions of V 2p and O 1s from V_2_C*T*_*x*_ before and after hydrolyzation at 300 °C. Based on the fitting parameters of previous work, the different V 2p_3/2_ peaks were assigned to V^2+^ (513.1 eV), V^3+^ (515.3 eV) and V^4+^ (516.3 eV).^31,41,42^ The paramagnetic splitting of V 2p is set to 7.2 eV with a split ratio of 0.5, which gives rise to the V 2p_1/2_ peaks at higher binding energies.^43^ The distribution of oxidation states in the pristine V_2_C*T*_*x*_ is in accordance with previous reports.^31,44,45^ Upon hydrolyzation, oxidation of the surface V atoms from 2+ to 4+ is evident, indicating possible oxide formation and/or change in the surface terminations. No contributions from V^5+^ (517.1 eV) in any of the spectra were detected, indicating that the hydrolyzation conditions are less oxidative than air.^30,31^ The deconvolution of the O 1s region is ascribed to vanadium oxides O–V (529.7 eV), O-terminated MXene as C–V–O (531.1 eV), and a combination of OH-terminated V_2_C*T*_*x*_ and adsorbed water as C–V–OH/H_2_O_ads_ (532.1 eV).^22,31,42,46,47^ The changes upon hydrolyzation indicate partial increase of surface oxides, with a reduction in the amount of OH-terminations and adsorbed water. This corresponds well with the changes in the V 2p region, and the XRD results presented in Figure 2, as well as with previous reports on the effect of V_2_C*T*_*x*_ annealing.^31^

In Table 1, the quantification of the chemical compositions of V_2_AlC, V_2_C*T*_*x*_, and V_2_C*T*_*x*_ after hydrolyzation at elevated temperatures is given. The EDS results are based on average values of several point scans and show a clear trend in reducing the concentration of F upon increasing the hydrolyzation temperature. The trend is supported by the EDS spectra from mapping presented in Figure 4c, demonstrating a significant reduction in the F Kα peak at 0.68 keV upon increasing the hydrolyzation temperature. The EDS results indicate a F reduction of around 2/3 in the bulk of V_2_C*T*_*x*_ particles hydrolyzed at 300 °C compared to pristine V_2_C*T*_*x*_. From Figure 4b, the reduction in F is even more significant at the outer surface of the particles as the F 1s peak is virtually absent after the hydrolyzation at 300 °C.

**Table 1:** EDS Data Averaged from Several Point Scans of V_2_AlC, Pristine V_2_C*T*_*x*_, and V_2_C*T*_*x*_ Hydrolyzed at Different Temperatures^a^

sample	V	Al	O	F	O (XPS)	F (XPS)
V_2_AlC	2	0.93	0.07	-		
V_2_C*T*_*x*_	2	0.03	0.27	1.00	1.31	0.54
Hyd100C	2	0.03	0.38	0.74		
Hyd200C	2	0.05	0.28	0.49		
Hyd300C	2	0.02	0.27	0.31	1.18	0.04
Hyd400C	2	0.09	0.35	0.15		
Hyd500C	2	0.03	1.06	0.03		

a The last two columns indicate the quantification obtained from the XPS fitting. All values are presented relative to one formula unit of V_2_C*T*_*x*_ (or a V amount of 2).

However, only minute changes in O-terminations are observed upon hydrolyzation. From Table 1, the EDS results indicate insignificant changes in the amount of O upon increasing the hydrolyzation temperature, where the O content remains stable at around 0.3 per unit formula of V_2_C*T*_*x*_. It should be noted that the quantification of V and O content by EDS is uncertain due to overlapping peaks, which is shown in Figure 4c. Nonetheless, the XPS results of the O 1s region also demonstrate a similar trend, where no obvious increase in the O-content can be seen after the hydrolyzation at 300 °C. From these results, it is therefore difficult to conclude whether the hydrolyzation has resulted in an increase of O-terminations or if F-terminations are simply removed, resulting in the formation of nonterminated V_2_C. Although formation of V_2_C does not match well with the increased oxidation state of V, removal of terminations matches well with the 2θ shift in the XRD results (Figure 2b), as V_2_C would have a smaller interlayer spacing than terminated V_2_C*T*_*x*_. Additionally, it should be noted that from the theoretically calculated Δ*G*°(T) curves presented in Figure 1b, the formation of single terminated V_2_CO is the most favorable reaction, indicating that formation of a partly nonterminated phase (V_2_CO) is more favorable than forming two O-terminations (V_2_CO_2_). With that in mind, even though characterization of the surface terminations is challenging with respect to MXenes, a more thorough study would be needed to confirm the nature of the termination groups on hydrolyzation, and to better quantify the amount of O-terminations.

### Thermal Stability

**Figure 5:**
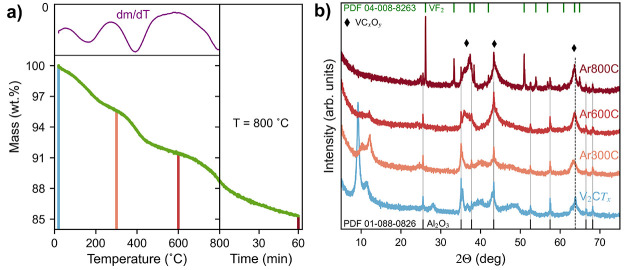
(a) TG curve of V_2_C*T*_*x*_ heated in Ar atmosphere up to 800 °C followed by 60 min dwelling. The inset shows differential mass loss during the heating step. (b) The resulting X-ray diffractograms after heating up to 300 °C, 600 °C and 800 °C, shown in (a). The dwelling times at the different temperatures were 60 min, and the dashed line in (b) indicates the position of the (110) MXene reflection at 63.8°.

Figure 5 shows the TG curve of V_2_C*T*_*x*_ in Ar atmosphere and the resulting X-ray diffractograms after various annealing temperatures. From the TG curve in Figure 5a, it is seen that the V_2_C*T*_*x*_ shows a continuous mass loss but with three significant mass loss regions at ∼80–280 °C, ∼320–500 °C and above ∼650 °C, matching well with previous reports.^12,30^ The initial mass loss is ascribed to desorption of physiosorbed water, which is supported by the 2θ-shift of the (002) V_2_C*T*_*x*_ reflections after annealing at 300 °C (Figure 5b). At higher temperatures (∼320–480 °C), the removal of chemisorbed water due to OH-terminations is suggested.^30,35^ However, the XRD data show that this temperature also results in a degradation of the MXene structure, as the (002) reflections of V_2_C*T*_*x*_ are significantly reduced after annealing up to 600 °C. Similar to hydrolyzation at 400 °C (Figure 2), the formation of broad reflections from oxycarbide (VC_*x*_O_*y*_) appear at this temperature. It is therefore suggested that the chemisorbed water may be required for the stability of the V_2_C*T*_*x*_ phase. At the third mass loss region (>650 °C), the MXene phase is fully degraded. According to Wu et al. only V_2_O_3_ and V_8_C_7_ phases were left after annealing up to 1000 °C.^30^ However, Figure 5b shows the presence of a VF_2_ phase after 1 h at 800 °C, indicating that some of the F content remained at this temperature. There is a chance that the discrepancies between these results might come from the initial O:F ratio obtained after etching, where Wu et al. showed a much higher O content than what is presented here (0.98:0.30 vs 0.27:1.00). This might have led to V_2_O_3_ being formed instead of VF_2_. Since Matthews et al. showed a significant mass loss between 800 and 1000 °C, it might also be that F is removed above 800 °C, resulting in the formation of only oxides and carbides.^35^

Comparing the results after annealing in pure Ar with the results from the hydrolyzation experiments (Figure 2), some differences with respect to the inclusion of water is observed. First, the remaining F phase after annealing at 800 °C in Ar (Figure 5b) indicates that the water vapor is essential for the removal of F during hydrolyzation, seeing that the F content was reduced to ∼0 after hydrolyzation at 500 °C (Table 1). Additionally, the splitting of the (002) reflections remains even after annealing in dry Ar, indicating that the chemisorbed water reacts similarly in wet and dry Ar. Moreover, the decomposition of V_2_C*T*_*x*_ starts at lower temperatures in the presence of water vapor, considering that the diffractogram after hydrolyzation at 400 °C resembles the one after annealing at 600 °C in Ar. However, even after annealing at 800 °C in dry Ar, the layered morphology of the particles remains (Figure S13), which is similar to what has been reported previously.^30,31^

### Electrochemical Properties

**Figure 6:**
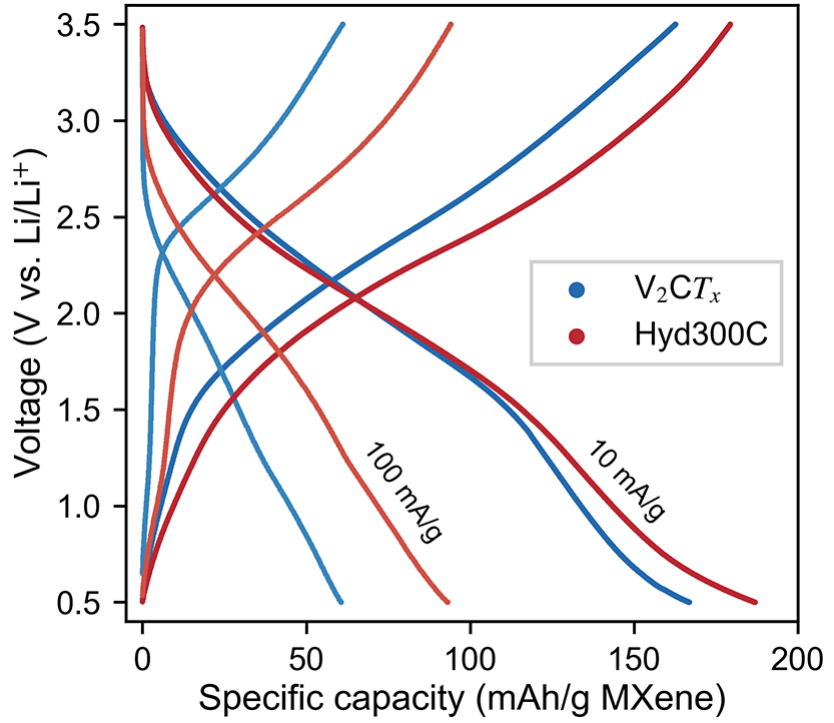
Voltage plots of two different cycles at two different current densities from V_2_C*T*_*x*_ electrodes before and after hydrolyzation at 300 °C.

The cycling results of V_2_C*T*_*x*_ electrodes in LiB half cells are presented in Figure 6. It shows voltage profiles from two cycles at two different current densities (10 and 100 mA/g) and demonstrates similar profiles for the pristine V_2_C*T*_*x*_ and the V_2_C*T*_*x*_ hydrolyzed at 300 °C. Both materials display generally sloped curves indicative of the pseudocapacitive storage mechanism of MXenes.^48,49^ With sloped plateaus at around 1.5–3 V, these voltage profiles are also similar to previously reported profiles for V_2_C*T*_*x*_, showing higher average voltages for V_2_C*T*_*x*_ MXene compared with other MXene compositions.^22,45,50^ Another interesting similarity with previous reports is the irreversible plateau observed at ∼1.6 V on the first discharge (Figure S16). As SEI formation is not expected to take place at such high voltages, it is possible that this plateau represents trapping of some Li-ions in V_2_C*T*_*x*_ on the first cycle.^51^ Nevertheless, these cycling results verify the presence of V_2_C*T*_*x*_ both before and after the hydrolyzation.

After comparing the electrochemical performance before and after hydrolyzation, only subtle differences are observed (Figure 6). This contrasts with some of the predicted changes from DFT calculations for changing termination groups. First, the average voltage was not increased after the removal of both F- and OH-terminations from hydrolyzation. This could be another indication of the hydrolyzation not resulting in any increase in the O-terminations, as the voltage then would have been expected to increase.^19,52^ Instead, this could also suggest the formation of nonterminated MXene. However, it might also be that the pseudocapacitive nature of the energy storage mechanism blurs out the effects of the intercalation voltages. Wang et al. presented similar voltages for V_2_C*T*_*x*_ etched in milder F-conditions (HCl and F-salts), resulting in fewer F-terminations than what is obtained by HF-etching.^45^ Although they demonstrated significantly higher capacities than by HF-etching, most of the capacity was obtained between 0.5 and 0.01 V. Above 0.5 V, their capacities were similar to the capacities obtained here.

The rate capability on the other hand was significantly improved after hydrolyzation of the MXene (Figure 6). Some of these changes can be related to the difference in loading (4.31 mg/cm^2^ for V_2_C*T*_*x*_ and 1.62 mg/cm^2^ for Hyd300C), but they cannot explain all the changes. Upon increasing the current density from 10 mA/g to 100 mA/g, the overpotential of the pristine V_2_C*T*_*x*_ increases more than for the hydrolyzed one. This change in polarization has previously been demonstrated for F-free Ti_3_C_2_*T*_*x*_ MXene^53^ and matches better with calculated results, as both bare and O-terminated V_2_C*T*_*x*_ have been shown to have lower migration barriers for Li-ions compared with OH- and F-terminated V_2_C*T*_*x*_.^24−27^ Notably, the reported F-free Ti_3_C_2_*T*_*x*_ also did not result in any significant change in intercalation voltage.

Comparing the first cycles of both cells, the irreversible plateau at ∼1.6 V is longer for the pristine V_2_C*T*_*x*_ than for the V_2_C*T*_*x*_ hydrolyzed at 300 °C (Figure S16). A possible explanation for this can be the reduction of the intercalated water between the MXene layers, where the pristine V_2_C*T*_*x*_ has the highest amount of intercalated water. Similar water reduction has previously been reported at comparable voltages in LiPF_6_ electrolytes with water impurities, where H_2_O is reduced into OH^–^ and H_2_ gas.^54,55^ However, if water reduction is the explanation of the irreversible plateau, it does not seem to have affected the long-term cycling performance of these electrodes significantly. Nonetheless, seeing that intercalated water remains in V_2_C*T*_*x*_ even after annealing in dry Ar at 300 °C, further research should be directed toward understanding the effect of intercalated water in MXenes when cycling in nonaqueous electrolytes.

## Discussion

Among the possible post etch treatments of MXenes, the present investigation has demonstrated that hydrolyzation of V_2_C*T*_*x*_ at elevated temperatures in a continuous flow of humidified Ar(g) can reduce the concentration of F-terminations. Although there have been several reports on hydrolysis of the Ti_3_C_2_*T*_*x*_ MXene phase, most of them have been performed under closed conditions, building up significant partial pressures of the product gases resulting from the hydrolyzation reaction and thus limiting further reaction. To the authors’ knowledge, there has only been reported one attempt on hydrolyzation of MXene in a continuous flow of humidified inert gas; however, this was performed on Ti_3_C_2_*T*_*x*_, at moderate hydrolyzation conditions (1 h at 400 °C, with 25 °C water bath and 100 mL/min gas flow).^48^ Based on the results presented here, the application of hydrolysis using a continuous flow of Ar(g) with a high partial pressure of H_2_O(g) opens up for new possibilities for post etching treatments of MXenes beyond only V_2_C*T*_*x*_.^35,56^

Another way of circumventing F-terminations would be to synthesize the MXene in a F-free environment. For the Ti_3_C_2_*T*_*x*_ phase, there has been reported several F-free etching methods, such as hydrothermal etching in NaOH solutions, in anhydrous halogen solutions and by molten salt reactions in both halogens and Lewis acids.^7,53,57−59^ Although the latter method was used to replace Al by Zn from V_2_AlC in the formation of V_2_ZnC, they were unable to further separate V_2_CCl_2_ sheets because of the high bond strength of V–Zn.^58^ Homogeneously terminated MXenes can be prepared by the molten salt method, where successful formation of pure Br-, Cl-, I-, Te-, Se-, NH_2_-, S-, and O-terminations of both Ti_3_C_2_*T*_*x*_ and Nb_2_C*T*_*x*_ MXenes has been demonstrated.^7^ However, to the authors’ knowledge, there has not yet been reported any successful etching of V_2_C*T*_*x*_ using any of these methods. Until other etching methods are successfully implemented for V_2_C*T*_*x*_, post etching methods will still remain essential in order to control the surface chemistry of this MXene.

## Conclusions

In summary, we have demonstrated gas hydrolyzation as a new and efficient method to significantly reduce the number of F-terminations from multilayered V_2_C*T*_*x*_ MXene. DFT calculations demonstrated that several hydrolyzation reactions are possible and that a continuous gas flow during the hydrolyzation would be ideal in order to maintain a high enough ratio between water vapor and the gas products. The V_2_C*T*_*x*_ was dehydrated upon annealing in both dry and wet Ar gas, leading to the removal of intercalated water and OH-terminations up to ∼300 °C. Additionally, hydrolyzation at 300 °C resulted in a removal of F content by almost 70% from the bulk of V_2_C*T*_*x*_ particles, thus demonstrating gas hydrolyzation as the most efficient post etching method for bulk F removal to date. However, at hydrolyzation temperatures above 300 °C, the MXene phase started to decompose, and a hydrolyzation temperature of 500 °C resulted in a complete transformation into V_2_O_3_ and C. In dry Ar, the decomposition started at slightly higher temperatures and only rock salt vanadium oxycarbides and VF_2_ remained after annealing at 800 °C.

Although hydrolyzation resulted in a reduction of F-terminations, it did not result in any corresponding increase in O content of the MXene. Instead, formation of nonterminated V_2_C is proposed, which is supported by the electrochemical performance of the hydrolyzed V_2_C*T*_*x*_. In LiB half cells, a reduced polarization was observed after hydrolyzation at 300 °C, matching well with the predicted lower migration barriers of bare and O-terminated V_2_C*T*_*x*_. All in all, our results present a new method for post etch removal of F-terminations from MXenes and introduces new insights on the thermal stability of V_2_C*T*_*x*_ in a hydrated atmosphere.

## Methods

### Synthesis of MAX Phase

The V_2_AlC MAX phase was synthesized by a solid-state reaction of V (Sigma-Aldrich, 99.5%), Al (Alfa Aesar, 99.5%), and graphite (Timcal Timrex, 99.5%) powders in a molar ratio of 2:1.3:1. The powders were mixed by wet ball (YSZ) milling in isopropanol overnight, dried in a rotavapor (Büchi R210) and subsequently pressed into 1 g cylindrical pellets at 25 MPa. The pellets were annealed in flowing Ar atmosphere at 1500 °C with a heating rate of 5 °C/min and a dwelling time of 4 h in a tube furnace (Entech ETF 17). To prevent oxidation, the tube was flushed with Ar for 4 h before the heat treatment. The synthesized MAX phase powder was mortared manually in a steel mortar followed by planetary milling at 300 rpm for 10 h in isopropanol with WC milling balls and milling jar, to reduce the particle size and obtain a narrow particle size distribution prior to etching (Figure S3).

### Synthesis of MXene

The multilayered V_2_C*T*_*x*_ MXene particles were synthesized by slowly adding 2 g of the synthesized V_2_AlC MAX phase powder in a polypropylene beaker with 40 mL of a 48 wt % HF solution over the time of 15 min. Thereafter, the beaker was partly covered with parafilm and etched at room temperature for 72 h under constant stirring. After the etching, the remaining powder dispersion was washed several times by centrifugation in DI-water dispersions, until reaching a pH > 5. In the end, the remaining dispersion was vacuum filtered through a 0.22 μm pore sized PVDF filter paper, before the powder was vacuum-dried at 120 °C for 12 h.

### Hydrolyzation

The hydrolyzation of V_2_C*T*_*x*_ was performed by spreading out 0.1 g of the MXene powder over 1–2 cm^2^ in an alumina crucible boat before introducing it to a quartz tube furnace (Figure S1). The furnace was sealed and after a 2 h flushing step with a flow rate of Ar gas (99.999%) at 200 mL/min, the furnace was heated to a given temperature at a rate of 200 °C/h and dwelled for 15 h with the same gas flow. To saturate the annealing atmosphere with H_2_O after the flushing step, Ar gas was bubbled through a DI water container at 80 °C prior to entering the tube. With a saturated vapor pressure of 4.738 × 10^4^ Pa,^32^ the water content in the Ar/H_2_O mixture was 47%. The exhaust gas was bubbled through a solution of 1 M Ca(NO_3_)_2_ in order to prevent air leakage into the furnace and to capture HF formed during the hydrolyzation.

### Characterization Techniques

The phase purity and crystalline structure of the products were characterized by X-ray powder diffraction (XRD, Bruker D8 Focus Diffractometer) using a Cu Kα radiation source (λ = 0.15418 nm) and a 0.2 mm slit size. The XRD data were collected in a 2θ-range from 5 to 75° with a step size of 0.0143° and a 0.68 s step time. The Al_2_O_3_ residue obtained from the MAX phase synthesis was used as a reference for the measurements, adjusting and scaling the spectra relative to its (012) reflection located at 25.57°. Thermal stability (TGA) of the MXene was measured with a NETZSCH STA 449 F3 Jupiter analyzer by placing ∼15 mg of the powder in α-Al_2_O_3_ containers with lid, heating it up under an Ar flow of 30 mL/min and a heating rate of 5 °C/min from RT to 300, 600, and 800 °C, with a dwelling time of 1 h. The particle size of the MAX phase was determined by laser diffraction (PSD, Horiba Partica LA-960) dispersing the powder in isopropanol to prevent agglomeration. The surface morphology and microstructure were investigated by a field-emission scanning electron microscope (FESEM, Carl Zeiss Ag – ULTRA 55) using an acceleration voltage of 5–10 kV. Energy dispersive X-ray spectroscopy (EDS) was used to assess chemical composition using an XFlash 4010 X-ray detector and an acceleration voltage of 10–15 kV. To obtain quantitative results, the average values from five or more point scans were chosen and analyzed using the Bruker Esprit 1.9 software (Figure S10). X-ray photoelectron spectroscopy (XPS) was used for further information on chemical composition of the powder. The XPS samples were made by gluing the MXene powder to a Si wafer substrate using silver glue, and the measurements were performed under ultrahigh vacuum using a SPECS XR-50 X-ray source with a Mg anode and a VG ESCA MKIV with a CLAM4 analyzer. The satellite peaks stemming from the Mg Kα_3_ and Kα_4_ were removed from the spectra before further data analysis were completed (Figure S14). To compensate for the static charge of the sample, the Si_2p_ peak from the substrate (99.3 eV) was used as an internal reference.^43^ The fitting of the curves was completed in the Igor Pro 7 software, using a Shirley background, and the quantification was performed using known photoionization cross-section values.^60^ The vibrational properties were investigated by a WITec Alpha 300r Confocal Raman Microscope, using a 100× objective, a 532 nm Ar laser and a laser power below 0.8 mW to prevent oxidation of the material (Figure S6).

### Electrochemical Measurements

To assess the electrochemical performance of the materials, LiB half cells were prepared with the MXene as the working electrode. These electrodes were processed by mixing N-ethyl-2-pyrrolidone (NEP)-slurries with 10 wt.% PVDF binder, 10 wt.% carbon black as conductive additive and 80 wt.% of the active material (V_2_C*T*_*x*_ and V_2_C*T*_*x*_ hydrolyzed at 300 °C). First, the carbon black and active material were mixed for 10 min at 25 Hz in a shaker mill. Next, a premade PVDF-NEP solution was added before the slurry was further diluted with additional NEP to obtain a solid to liquid ratio of 1:6. The slurries were then mixed by continuous shaking at 15 Hz for 30 min with a shaker ball and drop cast onto precut circular Al current collectors. The electrodes were dried at room temperature in a fume hood overnight before being dried in vacuum at 60 °C for at least 4 h. This resulted in active material loadings of 1.6–4.3 mg/cm^2^. The electrodes were assembled into 2016-type coin cells in an argon-filled glovebox (O_2_ ≤ 0.1 ppm, H_2_O ≤ 0.1 ppm) using Li-foil as the counter electrode, glass microfiber (Whatman) as the separator, and 110 μL 1 M LiPF_6_ in ethylene carbonate and ethyl methyl carbonate with a volume ratio of 1:1 (EC/EMC 1:1) as the electrolyte. The assembled cells were galvanostatically cycled at various specific currents (10 mA/g-100 mA/g) in a voltage range of 0.5 to 3.5 V using a BioLogic BCS-805 cycler at a controlled temperature of 20 °C.

### Theoretical Calculations

Density functional theory calculations were done with VASP^61−64^ using the PBEsol functional^65−67^ and a plane-wave energy cutoff of 650 eV. Gamma-centered k-point meshes with ∼0.2 Å^–1^ spacings were used for solid structures. Geometries were relaxed until the forces on the ions were below 10^–4^ eV/Å to obtain ground state energies (E0(T = 0 K)). Vibrational properties of the solid were calculated with Phonopy,^68^ and those for gaseous species were calculated by standard statistical mechanics.^69,70^ Corresponding zero-point energies (ZPE) were calculated for the solids and taken from the NIST-CCCBDB database for gaseous species.^71^ The thermodynamic properties of chemical reactions were evaluated following ref (72). Pseudopotentials, Δ*G* curves for more reactions, and a full description of the computational workflow are given in the Supporting Information.

## Supporting Information Available

(PDF) The Supporting Information is available free of charge at https://pubs.acs.org/doi/10.1021/acsomega.2c02441.

Detailed description of the DFT workflow with pseudopotentials and thermodynamic results for additional reactions, schematic of the hydrolyzation setup, additional experimental data including X-ray diffractogram of V_2_AlC MAX phase before and after etching, PSD plots and SEM images from V_2_AlC before and after wet milling, Raman spectra, EDS spectra, full XPS spectra and satellite removal of O 1s and V 2p regions, galvanostatic cycling plots of first cycles, and SEM images of V_2_AlC and V_2_C*T*_*x*_ after the TG annealing in Ar (PDF)
