# Supplementary material for: Removing Fluoride-Terminations from Multilayered V2CTx MXene by Gas Hydrolyzation
Source: ACS Omega. 2022 Jun 24;7(27):23790–9. doi: 10.1021/acsomega.2c02441 (PMC9280772; doi:10.1021/acsomega.2c02441)
Supplement: Supplementary file 1 — ao2c02441_si_001.pdf [file ao2c02441_si_001.pdf]

Supplementary information:

Removing Fluoride-terminations from Multilayered V_2CT_x MXene by Gas Hydrolyzation

Frode Håskjold Fagerli,[a] Zhaohui Wang,[ab] Tor Grande,[a] Henning Kaland,[a] Sverre M. Selbach,[a] Nils Peter Wagner[ab] and Kjell Wiik*[a]

a. Department of Materials Science and Engineering, NTNU Norwegian University of Science and Technology, Sem Sælands vei 12, NO-7034 Trondheim, Norway.

b. SINTEF Industry, Richard Birkelands vei 3, NO-7034 Trondheim, Norway.

Details of DFT calculations and workflow on the thermodynamic property evaluation of the proposed hydrolysation reactions

Density functional theory (DFT) calculations were carried out using the projector augmented wave (PAW) method as implemented in the Vienna Ab initio Simulation Package (VASP),[1-4] using V_sv and standard C, O, H, F pseudopotentials, and PBEsol exchange-correlation functional.[5-7] Plane wave basis sets were used with a kinetic energy cutoff of 650 eV. Gamma-centered k-point sampling was employed with a spacing close to 0.2 $Å^{-1}$ along the 3 axes of the reciprocal unit cells for all structures, except that for the isolated gas molecules which were placed in a 15x15x15 Å box and calculated by 1x1x1 gamma centered k-point. Spin-polarization were considered for all compounds.

The computational workflow on the evaluation of the thermodynamic properties of the proposed hydrolyzation reactions in this study included a *geometry relaxation* with a tight electronic convergence criterion of 1 x 10^{-8} eV and forces on the ions of less than -1 x 10^{-4} eV/Å. Ground state energies (E0(T=0 K)) were calculated for the optimized structures. *Thermal properties*, namely phonon free energy (F(T)), entropy (S(T)) and the phonon contribution on internal energy (E(T)), of all solids (V_2CF_2, V_2CO_2, V_2CO, $V_2C(OH)_2$, V_2O_3, VF_2, and VC) were calculated based on the optimized structures using frozen phonon method within the open-source package Phonopy.[8] The corresponding entropy (S(T)) and enthalpy (H(T)) for gaseous species (F_2, O_2, H_2, H_2O, HF, CO_2) were evaluated by standard statistical mechanics.[9, 10] Zero-point energies (ZPE) were calculated by equation

$$ZPE = \frac{1}{2}\sum_{i=1}^{n} v_i \qquad (1)$$

where v_i is frequency. For solid compound, ZPE was E(T) at T→0 obtained from phonon calculations. v_i for gaseous species were taken from NIST- CCCBDB database.[11]

Thermodynamic properties of reactions (ΔG(T), ΔH(T) and ΔS(T)) were further evaluated according to an established method based on the ground state energy (E0(T=0 K), ZPE, thermal energies (F(T), E(T), S(T)) of each individual compound.[12] Temperature dependent H(T), at a given temperature was computed by a sum of the internal energy at zero Kelvin by DFT, plus ZPE and its contribution by phonon vibrations E(T). Explicitly, enthalpy was approximated by the following equations:

For solids:

$$H(T) = E0 + E(T) \quad (2)$$

where ZPE is E(T) at T = 0 K, and for gases:

$$H(T) = E0 + ZPE + aRT + \frac{5}{2}RT + \sum_{i=1}^{n} \frac{N_A h v_i}{e^{hv_i/kT-1}} \quad (3)$$

where a = 1 and 3/2 for linear and non-linear molecules respectively, N_A is Avogadro constant and k is Boltzmann constant. S(T) for solid compounds was obtained by phonon calculations and for gases were taken from NIST-JANAF thermochemical table.[13] As the thermal properties for each individual compound were obtained, the thermodynamic properties of the reactions could be evaluated by:

$$\Delta H(T) = H(T)_{products} - H(T)_{reactants} \quad (4)$$

$$\Delta S(T) = S(T)_{products} - S(T)_{reactants} \quad (5)$$

$$\Delta G(T) = \Delta H(T) - T\Delta S(T). \quad (6)$$

To calculate the partial pressure of HF gas at equilibrium, with a given water vapour pressure, the equation for Gibb's free energy was used with reaction coefficients given by the different hydrolysation reactions:

$$\Delta G^{\circ} = -RTln(K) = -RTln\left(\frac{p(\mathrm{HF})^x}{p(\mathrm{H_2O})^y}\right) \quad (7)$$

Table S1. Chosen DFT data for the reactions presented in Figure S11, being the change in ground state energy (ΔE0), the change in Zero-point energy (ΔZPE), and the enthalpy change (ΔH(T)) for the different reactions at three different temperatures: T = 0 K, 300 K and 600 K.

**Reaction**	**ΔE0 (eV)**	**ΔZPE (eV)**	**ΔH(0K) (kJ/mol)**	**ΔH(300K) (kJ/mol)**	**ΔH(600K) (kJ/mol)**
**Termination change**					
1. V2CF2 + 2H2O (g) = V2C(OH)2 + 2HF (g)	0.320	-0.025	28.475	26.791	30.674
2. V2CF2 + 2H2O (g) = V2CO2 + 2HF (g) + H2 (g)	0.621	-0.296	31.352	35.757	40.386
3. V2CF2 + H2O (g) = V2CO + 2HF (g)	0.368	-0.065	29.165	31.137	30.646
4. V2C(OH)2 = V2CO2 + H2 (g)	0.301	-0.271	2.8765	8.967	9.713
**Decomposition of MXene**					
5. V2C(OH)2 + H2O (g) = V2O3 + C + 2 H2 (g)	0.616	-0.427	18.273	27.071	30.895
6. V2CF2 + 1.5 H2O (g) = 0.5 V2O3 + VC + 2HF (g) + 0.5H2 (g)	0.974	-0.205	74.271	79.133	81.518
7. V2CO + 0.5 H2O (g) = 0.5 V2O3 + VC + 0.5 H2 (g)	0.607	-0.139	45.105	47.997	50.872
8. V2CF2 = VC + VF2	0.365	-0.019	33.383	33.776	34.262
9. V2C(OH)2 + 3H2O (g) = V2O3 + CO2 (g) + 4H2 (g)	2.107	-0.855	120.730	135.410	144.930
10. V2CO2 + 3H2O (g) = V2O3 + CO2 (g) + 3H2 (g)	1.806	-0.584	117.850	126.440	135.220
11. V2CO + 4H2O (g) = V2O3 + CO2 (g) + 4H2 (g)	2.059	-0.815	120.040	131.060	144.960
12. V2CF2 + 5H2O (g) = V2O3 + CO2 (g) + 2HF (g) + 4H2 (g)	2.427	-0.880	149.200	162.200	175.610

Additional figures and tables

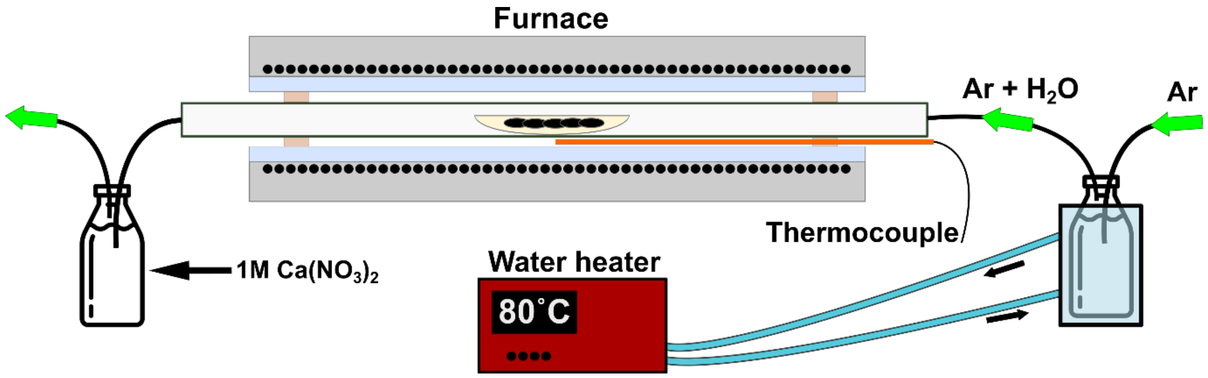

Figure S1. The setup of the hydrolysation furnace with a water heated gas bubble flask in front of the furnace to ensure high water vapour pressure inside the reaction tube. A solution of 1M Ca(NO$_3$)$_2$ was used at the end to bind potential HF gas from the hydrolysation reaction.

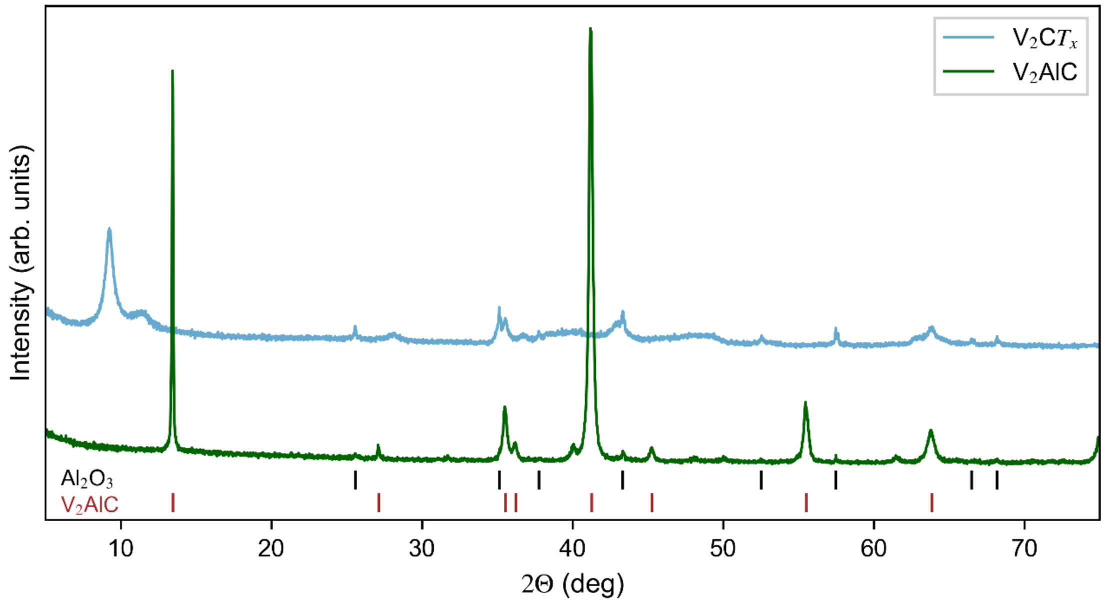

Figure S2. X-ray diffractogram of the V_2AlC *MAX phase after milling and the resulting* V_2CT_x *after etching of the milled MAX phase in 48 wt. % HF for 72 h. The marked* V_2AlC *reflections come from PDF 00-029-0101 and the Al2O3 reflections from PDF 01-088-0826.*

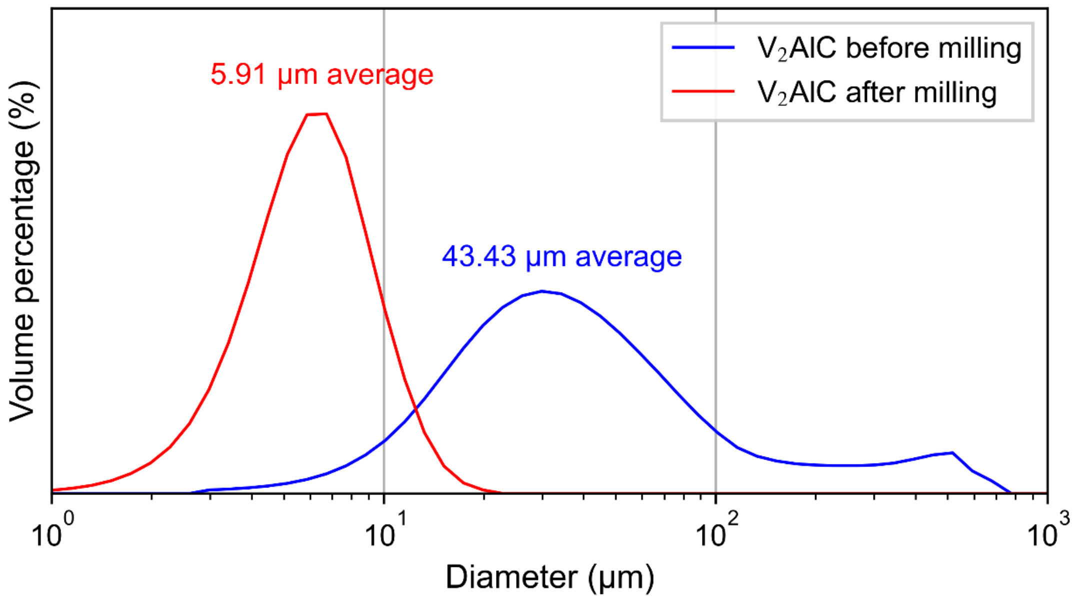

Figure S3. Particle size distribution, obtained by laser diffraction, of V_2AlC *MAX phase before and after wet milling for 10 hours at 300 rpm.*

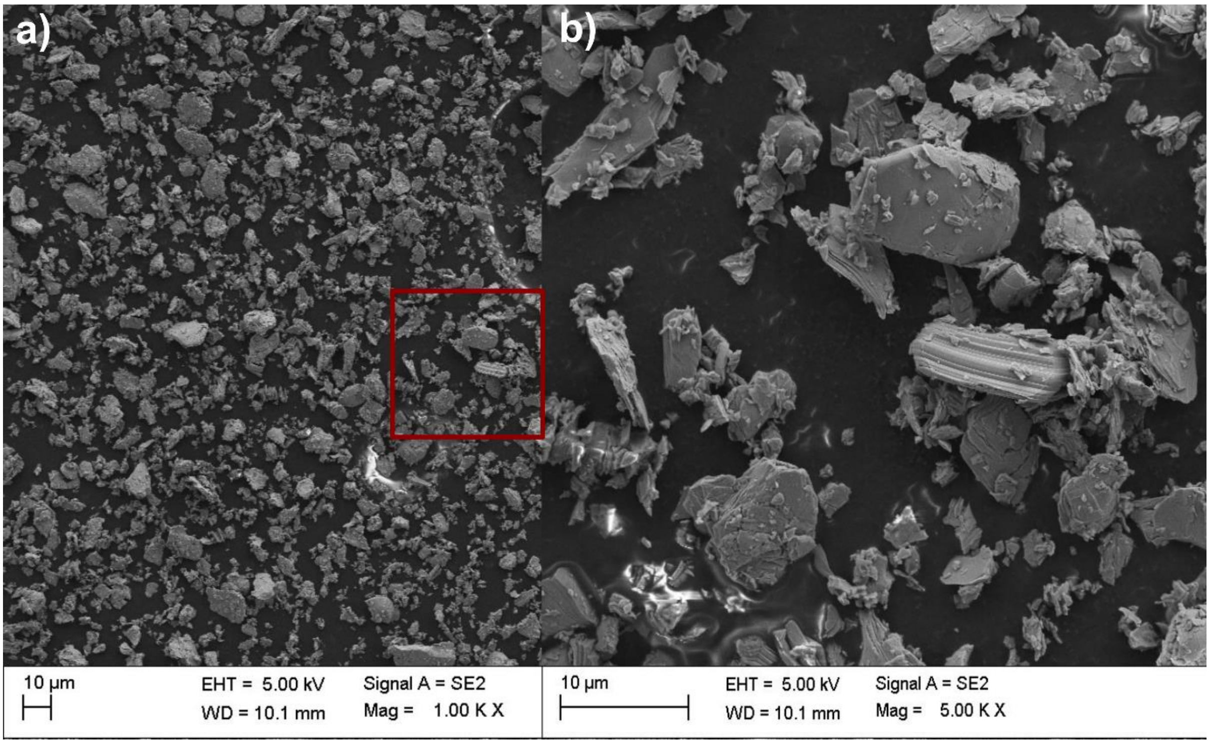

Figure S4. SEM images at 1k (a) and 5k (b) magnification of V_2AlC *after wet milling showing the particle size distribution and the variation in morphology of the particles. The area of figure (b) is marked in red in figure (a).*

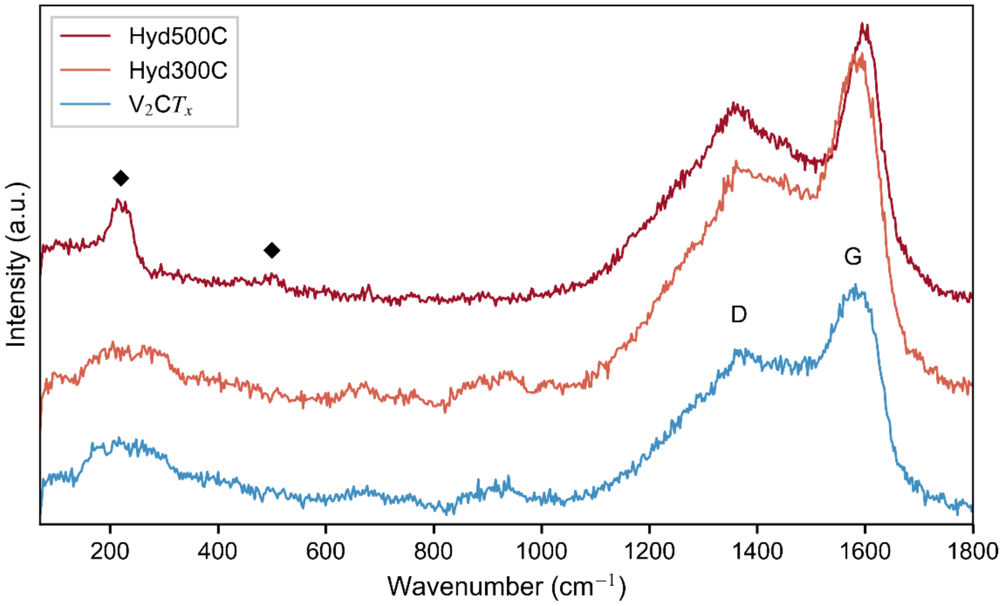

Figure S5. Raman spectra of V_2CT_x *before and after hydrolysation at 300 ˚C and 500 ˚C indicating the formation of* V_2O_3*, marked with diamonds, at the highest temperature.*[14] *MXene bands at around 220 cm*$^{-1}$*, 650 cm*$^{-1}$ *and 900 cm*$^{-1}$ *remain stable up to 300 ˚C, and the spectra partly resemble those presented in previous reports.*[15-17] *The presence of* C *D and G bands indicate that some amorphous C remain even after complete decomposition of the MXene phase. The presence of these carbon peaks, and a great variation of MXene peaks have previously been reported by Thakur et al..*[18]

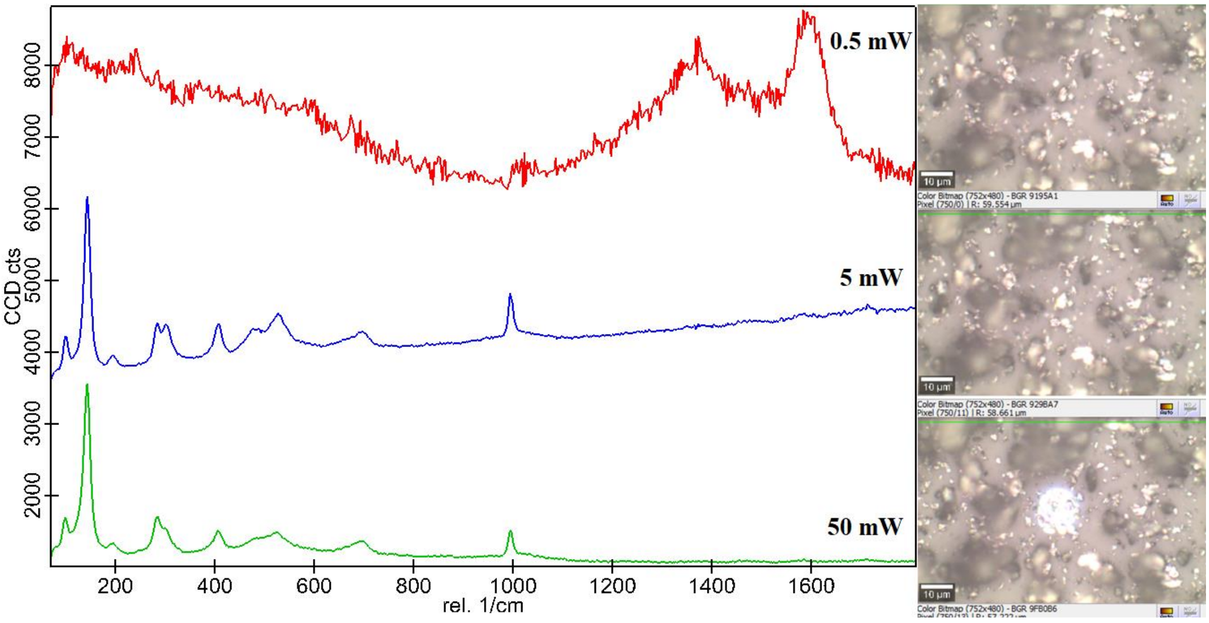

Figure S6. Raman spectra of V_2CT_x *hydrolysed at 500 ˚C at different laser powers. Initially there is only visible peaks related to C at 1400 and 1600 cm*$^{-1}$*. The peaks that arise at higher laser powers match ok with alpha-*V_2O_5*,*[19] *showing how the phase oxidizes and forms large white particles under the influence of the Raman laser at higher laser powers.*

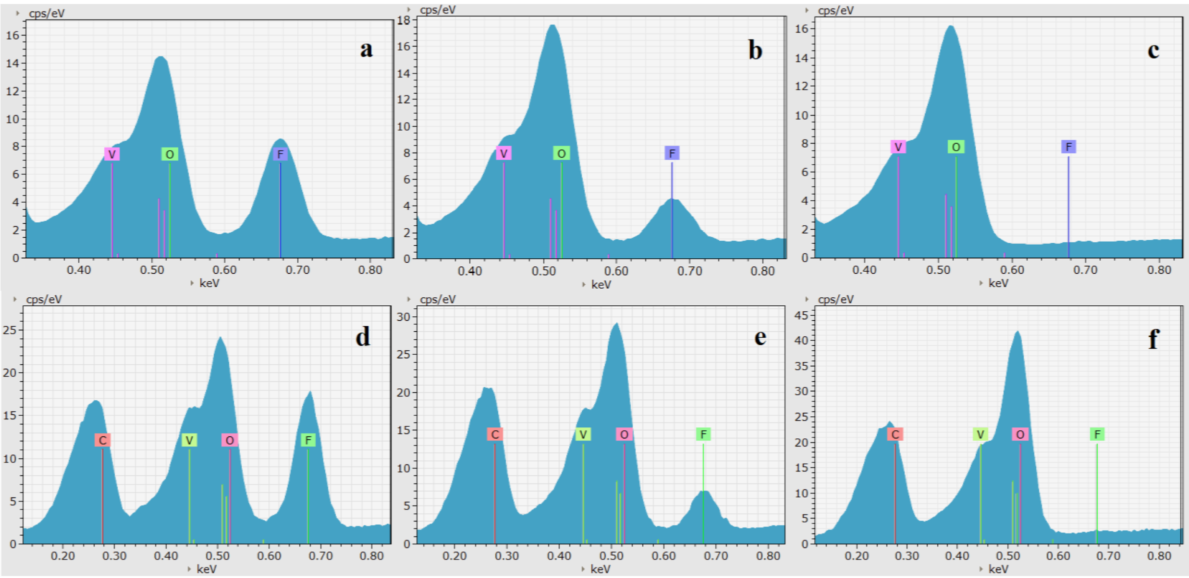

Figure S7. Showing the low energy region of EDS spectra obtained from mapping of pristine V_2CT_x (a), V_2CT_x hydrolysed at 300 ˚C (b) and 500 ˚C (c), and from a representative point scan measurement of pristine V_2CT_x (d), V_2CT_x hydrolysed at 300 ˚C (e) and 500 ˚C (f). The C peak is omitted from the mapping plots due to the high intensity coming from the carbon tape background. From these figures we see a clear trend in reduction of F from hydrolysation at increased temperature. Due to the overlap of the Lα1 (0.510 eV) and Lβ1 (0.518 eV) peak from V with the only EDS peak of O (Kα1, 0.525 eV), it is difficult to get any useful quantification data of the O content. The remaining V peaks are LL (0.446 eV), LE (0.454 eV) and Lβ3 (0.590 eV).

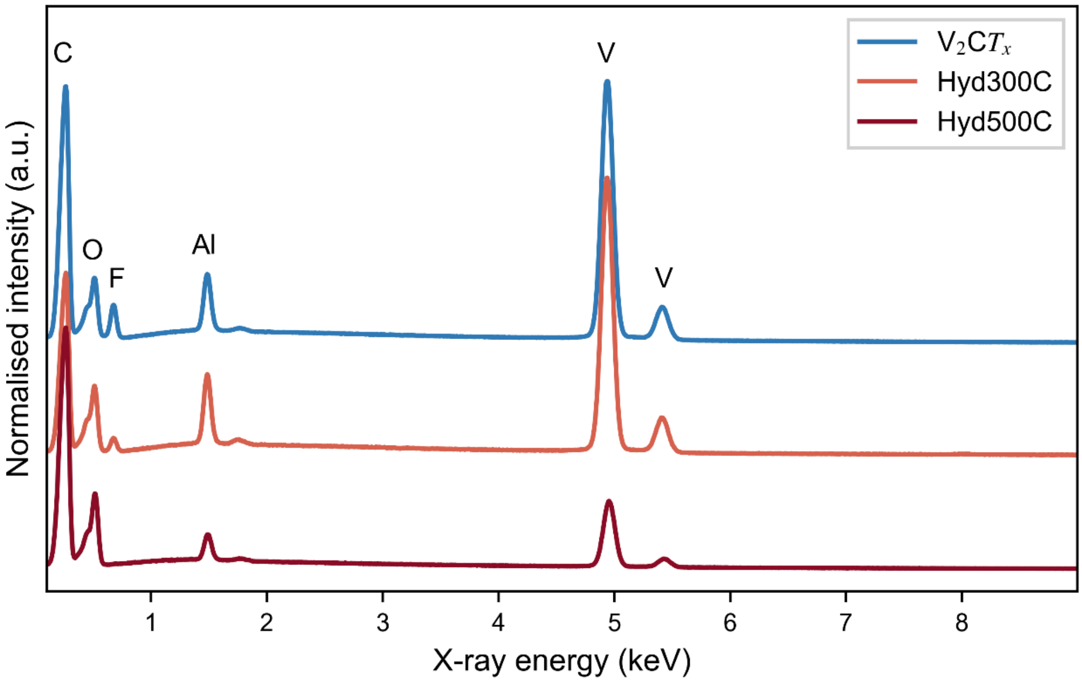

Figure S8. Full spectra of the EDS mapping of V_2CT_x presented in Figure 4. The intensity of the peaks at higher energy (Al and V) are lower for the powder hydrolysed at 500 ˚C due to a lower acceleration voltage (10 keV vs. 15 keV) of the electron beam used for the measurements. The peaks at lower energies (C, O and F) should not be influenced significantly by this change, as these X-rays do not live long enough to travel from deep within the particles. Al is present in these mapping spectra due to rests of Al_2O_3 particles from the MAX phase synthesis.

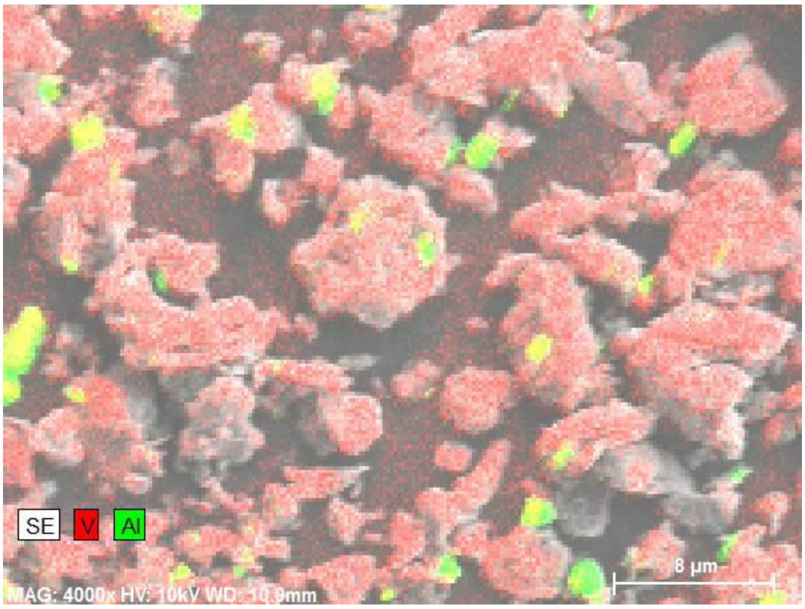

Figure S9. EDS mapping of the V_2CT_x *that is used for the results in Figure 4c and Figure S8. The green dots indicate several* Al *rich particles that represent* Al_2O_3*.*

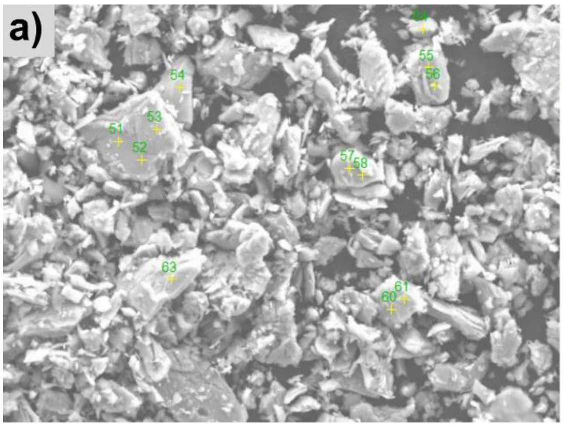

b)

Point scan	V	Al	C	O	F	Comment
51	56.85	27.19	12.73	2.76	0.07	Good
52	57.98	26.71	13.71	1.2	0.04	Good
53	55.61	27.26	15.38	1.15	0.27	Good
54	49.56	27.42	22.15	0	0.57	Good
55	60.08	26.7	12.33	0.49	0	Good
56	54.65	25.62	16.25	2.82	0.3	Good
57	63.17	23.63	7.12	5.64	0	Good
58	70.46	19.57	6.82	2.56	0	High tilt
60	80.27	9.51	3.01	7.21	0	High tilt
61	74.7	12.87	3.73	8.7	0	High tilt
63	50.98	27.07	16.56	4.34	0.67	Good
64	2.53	39.83	11.36	45.13	0.9	Al_2O_3

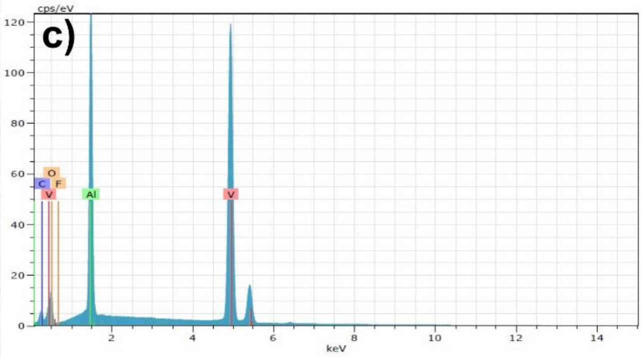

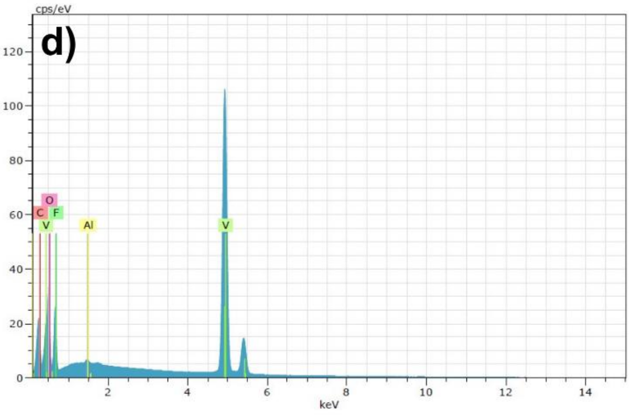

Figure 10 Demonstration of how the EDS average values were obtained, showing the points that were scanned from the V_2AlC *MAX phase (a), and the resulting atomic percentages of different elements for each point scan after analysis (b). In (b) it is commented which spectra were used for the average value, as some of the spectra were from* Al_2O_3 *particles or had very high uncertainties due to high tilt of the particles. In (c) and (d), representative point scan spectra from before (c) and after etching (d) are shown to visually demonstrate the removal of Al upon etching as presented in Table 1.*

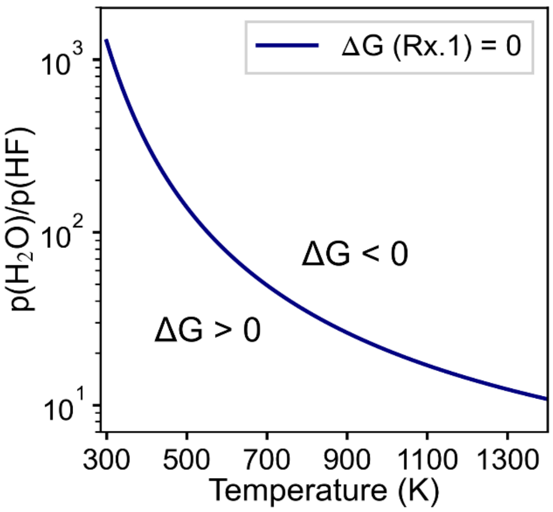

Figure S11. A curve indicating the partial pressure ratio between H_2O *and* HF *required to make reaction 1 in Fig. 1 have ΔG = 0. To the right of this line, this reaction would also be thermodynamically spontaneous.*

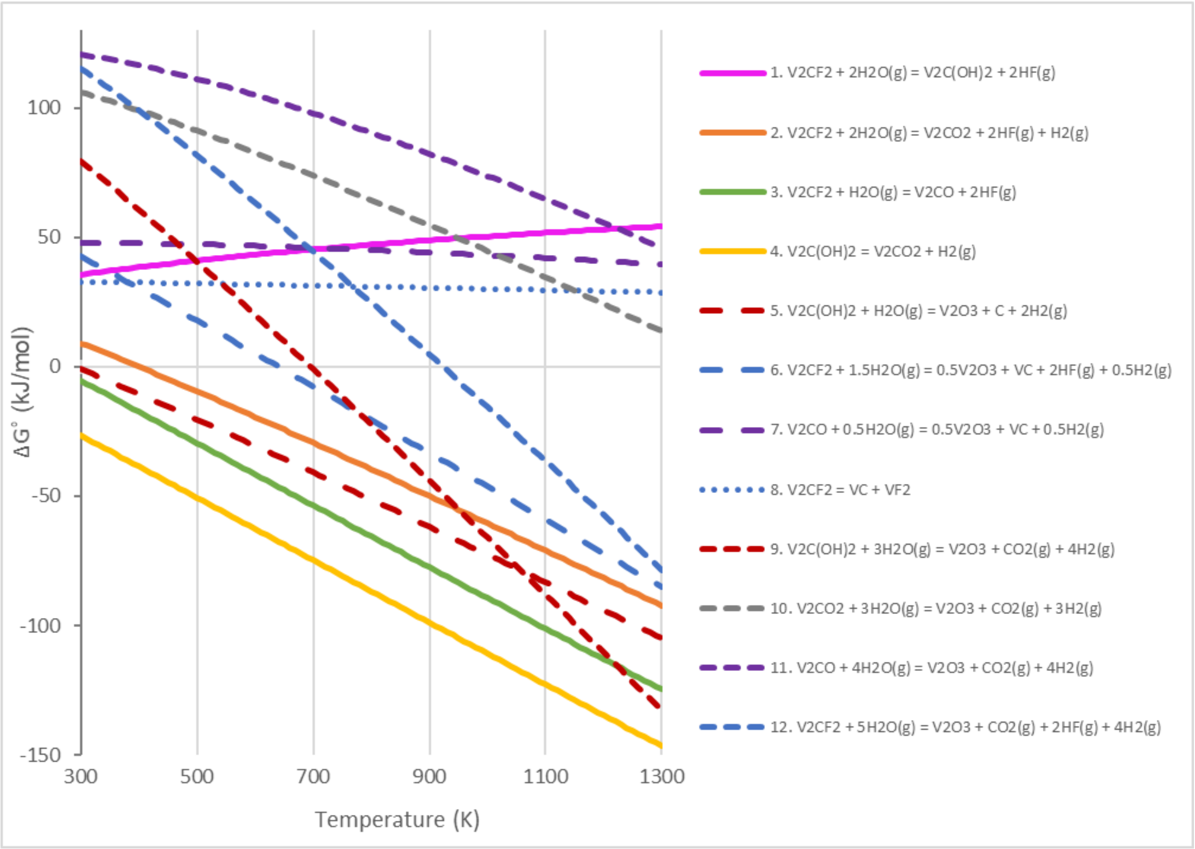

Figure S12. ΔG° as a function of temperature for several reactions of V_2CT_x*, including four termination group alternating reactions shown in solid lines (1-4), as well as various decomposition reactions (5-12) in dashed lines. The decomposition reactions are coloured by the termination of the reactant* V_2CT_x *phase. Reactions 1-3 are already presented in Fig. 1a.*

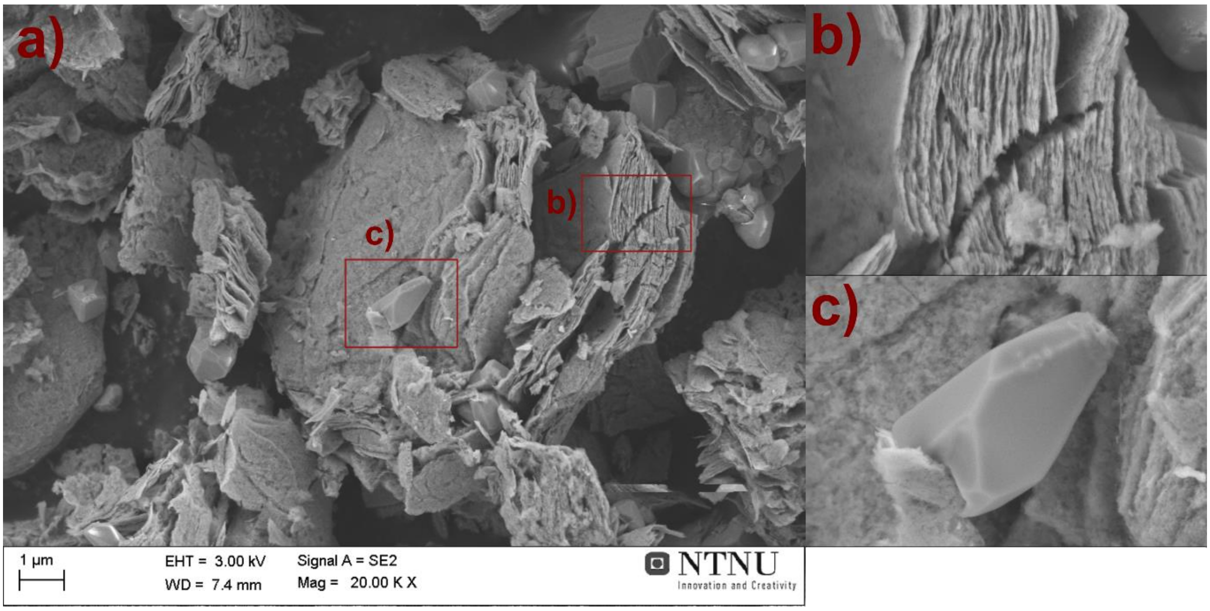

Figure S13. Overview SEM image of V_2CT_x *after TG annealing in* Ar *to 800 ˚C (a) where we see that the layered morphology of the particles remains (b) although we also see possible formation of crystalline* VF_2 *particles (c).*

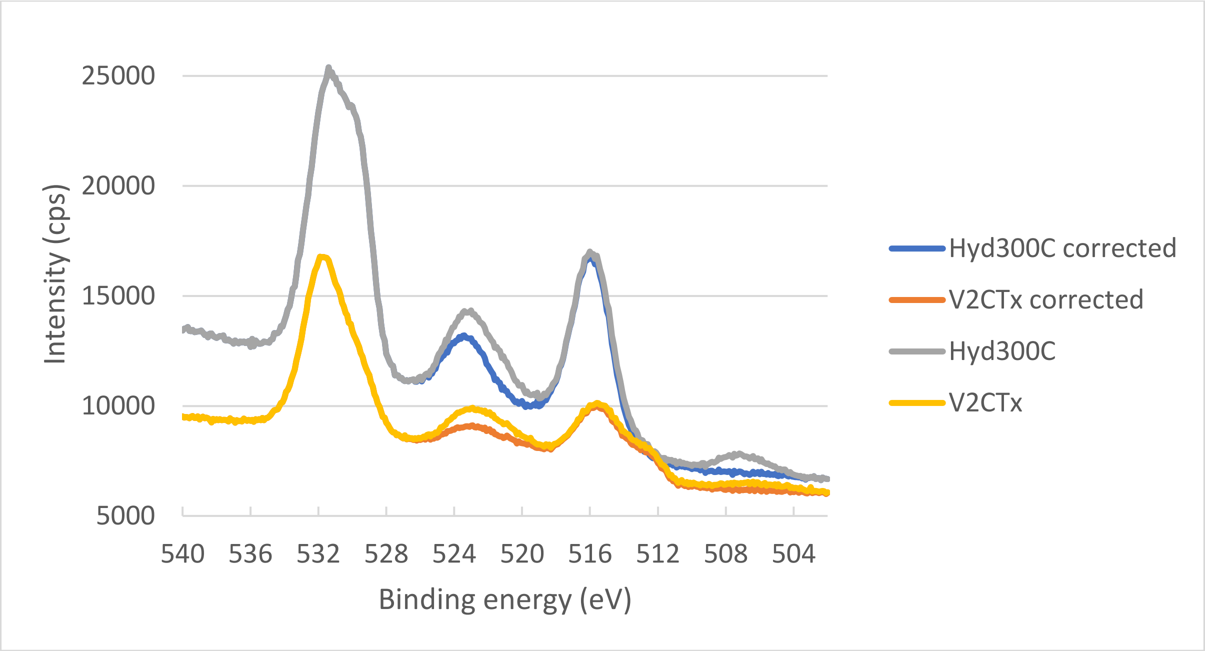

Figure S14. The XPS data of the V *2p and* O *1s region before and after removing the Mg Kα satellites for both pristine* V_2CT_x *and the MXene hydrolysed at 300 ˚C. The satellite free ("corrected") values were used in the XPS fitting of this region used in Figure 4.*

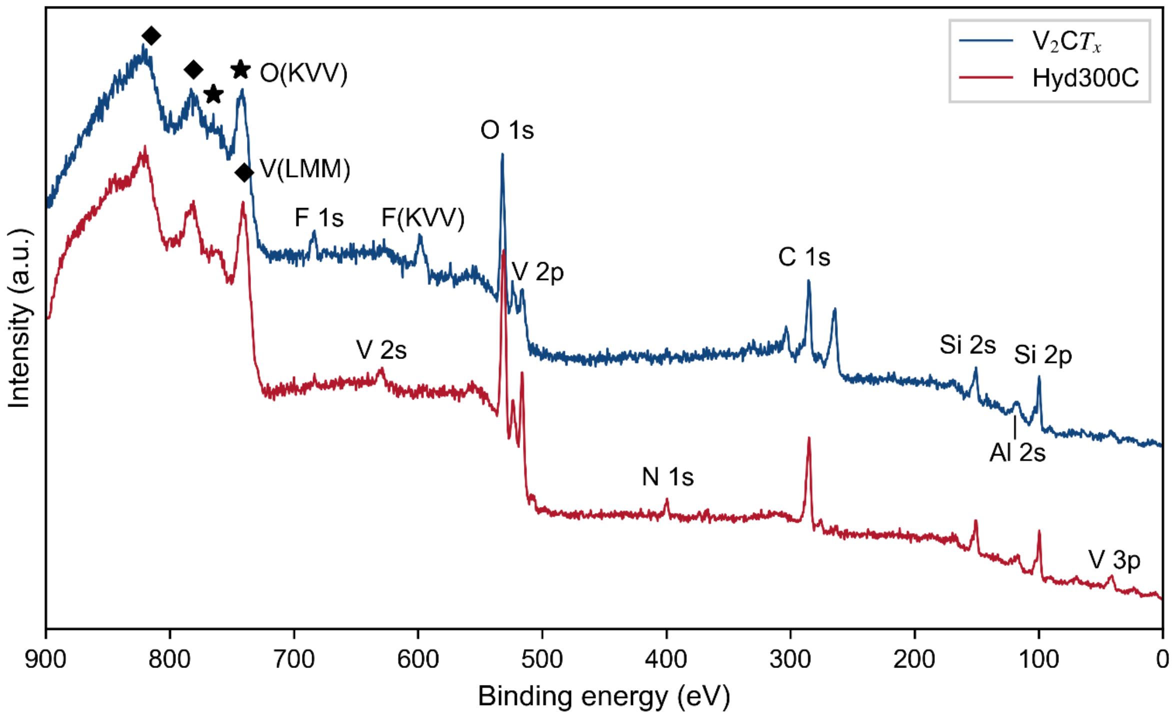

Figure S15. Full XPS spectra of V_2CT_x *before and after hydrolysation at 300 ˚C without the removal of Mg Kα satellites. The* Si *peaks come from the powder being dispersed on a* Si *wafer substrate and were used as internal reference. The peaks at around 265 eV and 305 eV in the pristine sample have not been assigned to any chemical component.*

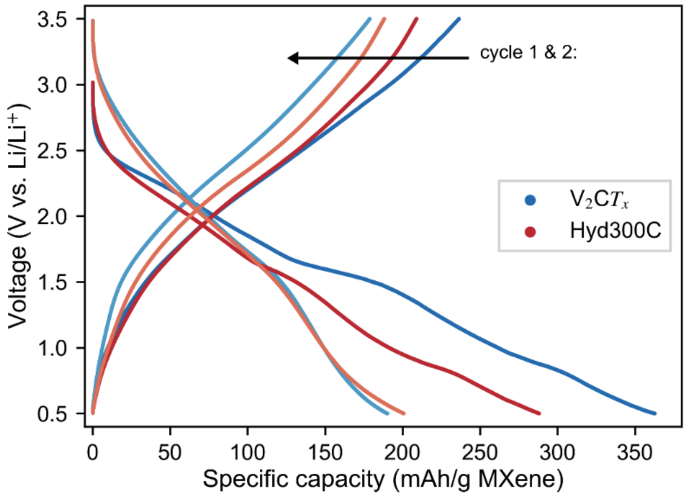

Figure S16. First two cycles from the galvanostatic cycling of LiBs with pristine V_2CT_x *and* V_2CT_x *hydrolysed at 300 ˚C. On the first cycle, there are two irreversible discharge plateaus, where the one at ~0.9 V can be assigned to SEI formation.*[20] *The plateau at 1.6 V might come from irreversible intercalation of Li-ions in between the MXene flakes.*

References:

(1) Kresse, G.; Furthmüller, J. Efficiency of ab-initio total energy calculations for metals and semiconductors using a plane-wave basis set. *Comput. Mater. Sci.* **1996**, *6*, 15-50.

(2) Kresse, G.; Furthmüller, J. Efficient iterative schemes for ab initio total-energy calculations using a plane-wave basis set. *Phys. Rev. B* **1996**, *54*, 11169-11186.

(3) Kresse, G.; Hafner, J. Ab initio molecular dynamics for liquid metals. *Phys. Rev. B* **1993**, *47*, 558.

(4) Kresse, G.; Hafner, J. Ab initio molecular-dynamics simulation of the liquid-metal–amorphous-semiconductor transition in germanium. *Phys. Rev. B* **1994**, *49*, 14251.

(5) Kresse, G.; Joubert, D. From ultrasoft pseudopotentials to the projector augmented-wave method. *Phys. Rev. B* **1999**, *59*, 1758.

(6) Blöchl, P. E. Projector augmented-wave method. *Phys. Rev. B* **1994**, *50*, 17953.

(7) Csonka, G. I.; Perdew, J. P.; Ruzsinszky, A.; Philipsen, P. H. T.; Lebègue, S.; Paier, J.; Vydrov, O. A.; Ángyán, J. G. Assessing the performance of recent density functionals for bulk solids. *Phys. Rev. B* **2009**, *79*, 155107.

(8) Togo, A.; Tanaka, I. First principles phonon calculations in materials science. *Scr. Mater.* **2015**, *108*, 1-5.

(9) Peter Atkins, P.; De Paula, J. Phys. Chem.; Oxford University Press, **2010**.

(10) Irikura, K. K. Appendix B Essential Statistical Thermodynamics. In ACS Symposium Series, Washington DC; **2001**.

(11) Johnson, R. D. NIST Computational Chemistry Comparison and Benchmark Database; http://cccbdb.nist.gov/, **2020**.

(12) Duan, Y.; Sorescu, D. C. Density functional theory studies of the structural, electronic, and phonon properties of Li2O and Li2CO3: Application to CO2 capture reaction. *Phys. Rev. B* **2009**, *79*, 014301.

(13) M. W. Chase and National Institute of Standards and Technology (U.S.), NIST-JANAF thermochemical tables, Am. Chem. Soc. & Am. Inst. Phys., 4th edn., 1998.

(14) Tatsuyama, C.; Fan , H. Y. Raman scattering and phase transitions in V2O3 and (V1−xCrx)2O3. *Phys. Rev. B* **1980**, *21*, 2977-2983.

(15) Narayanasamy, M.; Kirubasankar, B.; Shi, M.; Velayutham, S.; Wang, B.; Angaiah, S.; Yan, C. Morphology restrained growth of V2O5 by the oxidation of V-MXenes as a fast diffusion controlled cathode material for aqueous zinc ion batteries. *Chem. Commun.* **2020**, *56*, 6412-6415.

(16) Chen, J.; Chen, K.; Tong, D.; Huang, Y.; Zhang, J.; Xue, J.; Huang, Q.; Chen, T. CO2 and temperature dual responsive "Smart" MXene phases. *Chem. Commun.* **2015**, *51*, 314-317.

(17) Jastrzębska, A. M.; Scheibe, B.; Szuplewska, A.; Rozmysłowska-Wojciechowska, A.; Chudy, M.; Aparicio, C.; Scheibe, M.; Janica, I.; Ciesielski, A.; Otyepka, M.; Barsoum, M. W. On the rapid in situ oxidation of two-dimensional V2CTz MXene in culture cell media and their cytotoxicity. *Mater. Sci. Eng. C* **2021**, *119*, 111431.

(18) Thakur, R.; VahidMohammadi, A.; Moncada, J.; Adams, W. R.; Chi, M.; Tatarchuk, B.; Beidaghi, M.; Carrero, C. A. Insights into the thermal and chemical stability of multilayered V2CTx MXene. *Nanoscale*, **2019**, *11*, 10716-10726.

(19) Shvets, P.; Dikaya, O. ; Maksimova, K.; Goikhman, A. A review of Raman spectroscopy of vanadium oxides. *J. Raman Spectrosc.* **2019**, *50*, 1226-1244.

(20) Xu, K., Nonaqueous Liquid Electrolytes for Lithium-Based Rechargeable Batteries. *Chem. Rev.* **2004**, *104*, 4303-4418.
